# Epithelioma of the Tongue in Women

**Published:** 1904-11-05

**Authors:** 


### EPITHELIOMA OF THE TONGUE IN WOMEN.

Mr. Charles Keyser[1] has collected ten unpublished cases of this disease from the records of the Cancer and St. George's Hospitals. He finds that women supply 8 09 per cent. of the total number of cases of carcinoma of the tongue which he has investigated in the records of four London hospitals. The age incidence does not differ appreciably in the two sexes; 55 years is about the average. Cases have been described in young women of 19 to 24 years. When discussing causation one or two interesting points are noticeable. Leukoplakia is commonly the precancerous stage, but syphilis is by no means a constant precursor and excessive smoking is certainly an uncommon predisposing cause. In Wales, where women smoke clay pipes, cancer of the tongue is rare. In Ireland, also, there seems no reason to suggest that smoking is an etiological factor in these cases.[2] All women in Finisterre smoke short pipes, but epithelioma of the tongue is unknown among them. Rough and carious teeth, badly fitting or broken tooth plates are the most usual irritants. The appearance, situation, course, diagnosis, prognosis, and treatment of the disease in women do appear to differ from those in man.

[1] Lancet, Sept. 17, 1904. [2] Barker, Practitioner, 1903.

